# Vegetation Dynamics in a Loess Grassland: Plant Traits Indicate Stability Based on Species Presence, but Directional Change When Cover Is Considered

**DOI:** 10.3390/plants11060763

**Published:** 2022-03-13

**Authors:** Péter Csontos, Júlia Tamás, Zsófia Kovács, Judit Schellenberger, Károly Penksza, Tibor Szili-Kovács, Tibor Kalapos

**Affiliations:** 1 Institute for Soil Sciences, Centre for Agricultural Research, Herman O. út 15, 1022 Budapest, Hungary; schellenberger.judit@atk.hu; 2 Department of Botany, Hungarian Natural History Museum, Könyves K. krt. 40, 1087 Budapest, Hungary; tamas.julia@nhmus.hu; 3 Doctoral School of Horticultural Sciences, Hungarian University of Agriculture and Life Sciences, Villányi út 29-43, 1118 Budapest, Hungary; kovacs.zsofi@atk.hu; 4 Plant Protection Institute, Centre for Agricultural Research, Nagykovácsi út 26-30, 1029 Budapest, Hungary; 5 Department of Botany, Hungarian University of Agriculture and Life Sciences, Páter Károly u. 1, 2100 Gödöllő, Hungary; penksza.karoly@uni-mate.hu; 6 Department of Plant Systematics, Ecology and Theoretical Biology, Loránd Eötvös University, Pázmány P. sétány 1/C, 1117 Budapest, Hungary; tibor.kalapos@ttk.elte.hu

**Keywords:** chorological spectrum, permanent plots, protected grassland, Raunkiaer’s life-forms, Shannon diversity, species turnover, vegetation turnover

## Abstract

This article evaluates the three-year vegetation dynamics of a species rich, protected steppe grassland on loess where no grazing occurred for decades at Bicske, Central Hungary. A detailed coenological survey of vascular vegetation was conducted in four permanent plots of 16 m^2^ each from 2018 to 2020. Raunkiaer’s life-forms, distribution range, and thousand-seed weight of species were evaluated. Shannon diversity and turnover rates for the species and the vegetation were also determined for each plot. In total, 108 vascular plant species were detected. The results indicate grassland stability when plant traits spectra were based on species presence data, but directional change if species cover values were used to weight trait categories. During the three years of the study, chamaephytes decreased and woody species increased their contribution for the Raunkiaer’s life-forms, while the cosmopolitan group has steadily lost its significance for distribution range types. Shannon diversity varied between 2.46 and 3.18 among plots (based on natural logarithm) and remained statistically unchanged through time. Average species turnover rates were 14.18% for 2018/19 and 17.52% for 2019/20, whereas corresponding values for vegetation turnover rates were 25.83% and 23.28%. Vegetation turnover rate was significantly higher than the species turnover rate.

## 1. Introduction

Steppe grasslands on loess are among the most species rich vegetation types of Hungary, harbouring several rare and endangered species. Before the advent of industrial agriculture, these grasslands covered large areas on the Hungarian Plain and the surrounding foothills [1]. By now, this vegetation type had shrunk to a fraction of its former territory, surviving in isolated fragments mostly on terrain unsuitable for mechanized agriculture, like steep slopes and narrow land stripes along motorways or railway lines, whereas some other patches were spared on prehistoric earthworks (kurgans, mottes, ramparts) considered as cultural heritage sites [2,3]. Therefore, each still existing steppe grassland patch on loess is of high botanical and nature conservation values today [4,5].

These grasslands understandably attracted the interest of botanical researchers. The relic patches of loess grassland vegetation played an important role in the reconstruction of the potential vegetation map of Hungary [6,7]. Due to their textural and structural complexity as well as historical importance, plant communities on loess soils were also frequent subjects of archaeobotanical and vegetation dynamical studies abroad [8,9,10,11,12]. In Hungary, loess vegetation was also intensively studied, led by professorFekete, a prominent plant ecologist, in the second half of the 20th century [13,14,15,16]. Further research focused on soil respiration and soil water content in connection with vegetation cover [17], as well as on the effect of indigenous fossorial mammals on the species composition of loess grasslands [18].

In general, biodiversity has long been shown to be influenced by environmental factors [19]. Besides studies on the effects of abiotic factors on biodiversity [20,21,22], the biotic environment has significant effects on biodiversity as well [23]. Interaction of biotic impact in combination with environmental conditions on biodiversity was also reported [24]. Documented examples prove that high diversity of one biotic component has supportive effect in increasing the diversity of another biotic component of the environment. In a review of 320 case studies, Castagneyrol and Jactel [25] convincingly demonstrated that the diversity of arthropods, birds, and mammals significantly increased with plant diversity regardless of species’ habitats. Multiple pieces of evidence also support the interaction between aboveground and belowground diversity via grazing the standing vegetation, individual plant species effects, plant species and functional type diversity, mixing of plant litter types, and other reasons [26,27]. However, there are difficulties in detecting these interactions mainly because soil organisms are much lesser known than aboveground biota; therefore, species-level comparisons are practically impossible in most cases [28]. Novel molecular genetic techniques like metagenomics provide a solution for this problem.

Our work is part of a multidisciplinary research on loess substrate that aims at uncovering the diversity of the soil microbiome and the coexisting macroscopic biota with particular interest in how various ways of farming influence diversity relative to that of a reference natural grassland.

Although the reference grassland can be considered as a stable vegetation, changes in its structure and diversity may occur due to the abandonment of past grazing, climate change, nitrogen deposition and other causes may also affect grasslands [29]. Tilman’s resource–ratio hypothesis model is among others the most likely to operate in this case [30,31].

This paper reports on the phytocoenological survey of the reference natural steppe grassland on loess conducted through three years. We give a detailed description of the plant community with special attention to vascular plant diversity, and also evaluate its change during the three years by using a plant functional trait approach.

## 2. Results

In total, 108 vascular plant species were detected in the plots. The highest number of species (71) was found in Plot-1 in 2018, whereas the lowest (39) was recorded in Plot-2 in 2019. Average and median number of species were 54.9 (SD = 10.3) and 52.5, respectively. The most abundant species were in decreasing order: *Brachypodium pinnatum*, *Festuca rupicola*, *Filipendula vulgaris*, *Euphorbia pannonica, Teucrium chamaedrys, Galium verum, Carex michelii* and *Salvia pratensis*. The complete list of species with their cover values are shown in the Appendix A, Table A1.

**Figure 1:**
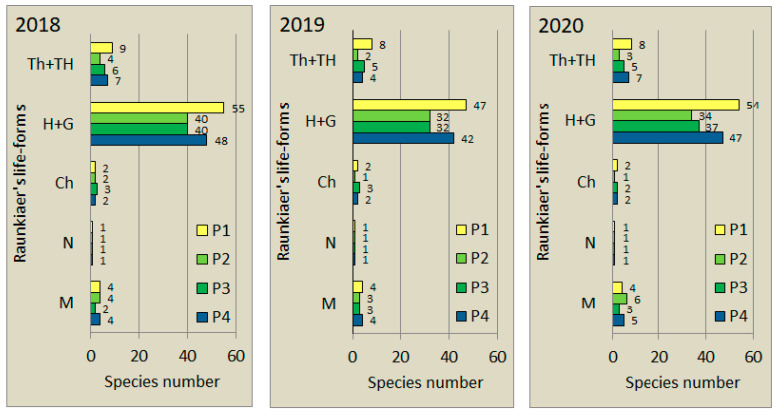
Life-form spectra of species in the four plots (P1–P4) of “Pócalja” Natura 2000 loess grassland (Central Hungary), in years 2018, 2019 and 2020. Th = therophytes, TH = hemitherophytes, H = hemicryptophytes, G = geophytes, Ch = chamaephytes, N = nanophanerophytes, M = phanerophytes.

Distribution of species according to Raunkiaer’s life-form categories in the three study years is shown in Figure 1. The groups of hemicryptophytes and geophytes (H+G) are dominant, followed by the group of annual and biennial species (Th+TH), whereas phanerophytes (M) has subordinate contribution throughout the three years. Chamaephytes and nanophanerophytes are practically negligible as they are represented by only 1–3 species.

**Figure 2:**
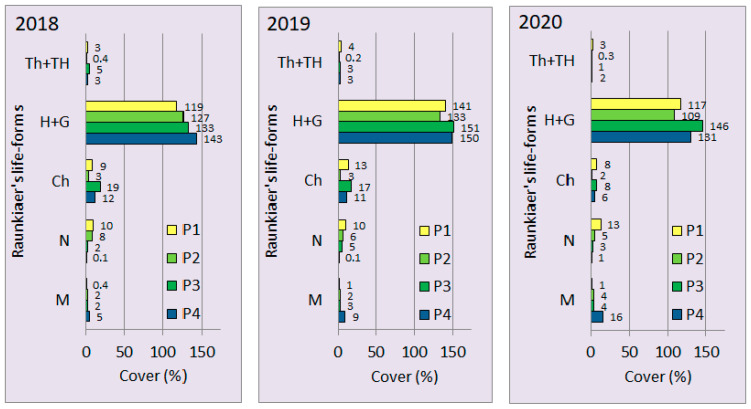
Life-form spectra based on species’ cumulative percentage cover values in the four plots (P1–P4) of “Pócalja” Natura 2000 loess grassland (Central Hungary), in years 2018, 2019 and 2020. Th = therophytes, TH = hemitherophytes, H = hemicryptophytes, G = geophytes, Ch = chamaephytes, N = nanophanerophytes, M = phanerophytes.

Considering the life-form spectra based on cover values, the H+G group maintained its overwhelming dominance in every year. At the same time, the least competitive species has lost some of their importance (Th+TH group), whereas chamaephytes decreased and woody species (N and M) increased their contribution in the vegetation of the grassland (Figure 2). Based on the chi-squared test performed on the merged cover data of the four plots, a significant shift appears in the life-form distribution over the three years (Chi^2^ = 16.183, *p* = 0.03983, d.f. = 8). According to the residuals, changes in the cover of chamaephytes (Ch) and phanerophytes (M) are mainly responsible for the shift in the spectrum.

**Figure 3:**
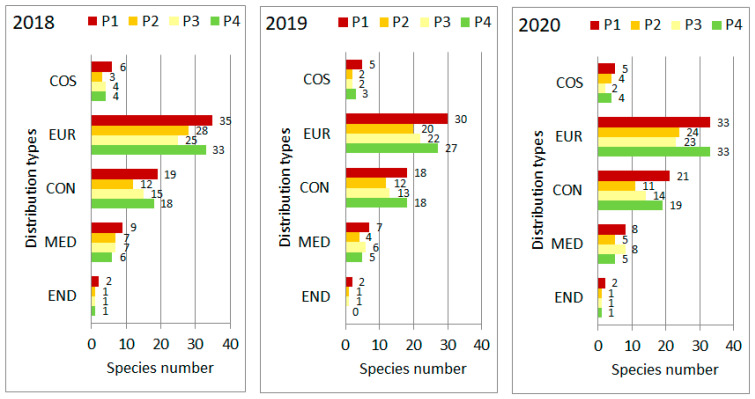
Share among distribution range types of species in the four plots (P1–P4) of “Pócalja” Natura 2000 loess grassland (Central Hungary), in years 2018, 2019 and 2020. COS = cosmopolitan, EUR = European, CON = continental, MED = Mediterranean, END = endemic.

According to chorological type of species, the European species are dominant followed by those of continental species (Figure 3). The ratio of species among the distribution range types remained basically similar during the study.

**Figure 4:**
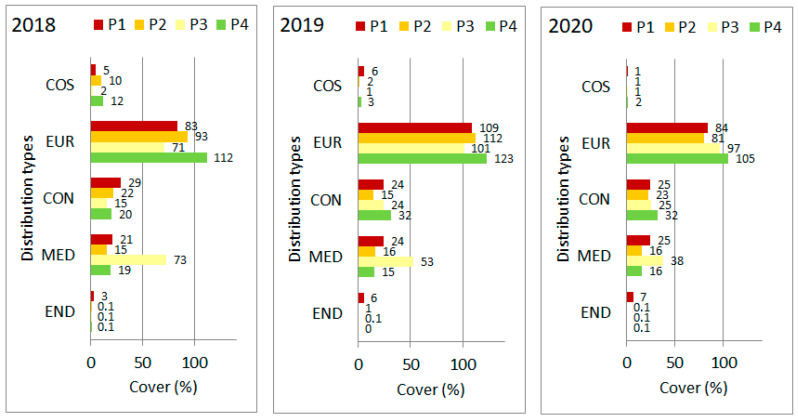
Share among distribution range types based on species’ cover values in the four plots (P1–P4) of “Pócalja” Natura 2000 loess grassland (Central Hungary), in years 2018, 2019 and 2020. COS = cosmopolitan, EUR = European, CON = continental, MED = Mediterranean, END = endemic.

When the distribution among range types was based on species cover values, the homogeneity test which was performed on the merged cover data of the four plots revealed highly significant differences among years (Chi^2^ = 34.251, *p* < 0.0001, d.f. = 8). The significant differences are mainly due to the annual fluctuations of some chorological groups. This can be observed even in the dominant European (EUR) group. The only significant one-way change was shown by the cosmopolitan (COS) group, which has steadily lost its significance during the years of observation (Figure 4).

**Figure 5:**
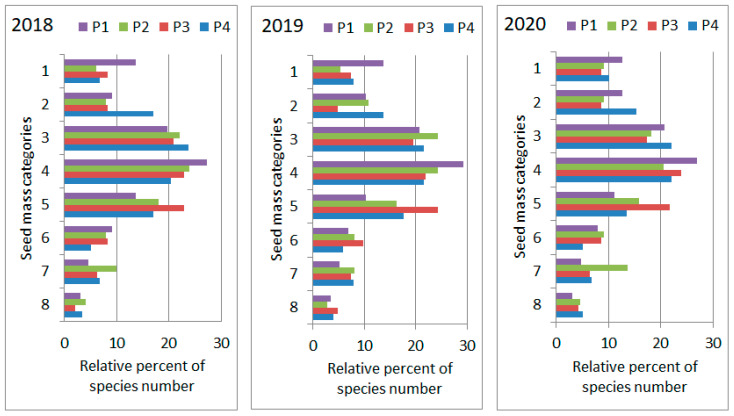
Distribution of species along seed weight categories in the four plots (P1–P4) of “Pócalja” Natura 2000 loess grassland (Central Hungary), in years 2018, 2019 and 2020.

The species pool in each of the four plots of the loess grassland was also analyzed by their thousand-seed weight. Relative representation of the eight seed weight classes was depicted based on yearly vegetation surveys from 2018 to 2020 (Figure 5). The seed weight spectra of the three study years proved to be statistically identical in a homogeneity test (Chi-square: 17.923; *p* = 0.9848). However, the loess grassland’s seed weight spectra differed significantly from that of the Hungarian flora (Chi-square: 120.03; *p* < 0.0001). The most striking difference was the over-representation of the intermediate seed weight categories (3, 4 and 5) in the loess grassland.

Species-abundance diversity values are summarized in Table 1. During the three years, the diversity of the grassland remained statistically unchanged (*p* = 0.6528 according to Friedman’s nonparametric repeated measures ANOVA). Average diversity (*n* = 12) was 2.857 (±0.20); however, the values fluctuated slightly, the highest value was 3.18 in Plot-4 and the lowest was 2.46 in Plot-2, observed in 2018 and 2019, respectively.

**Figure 6:**
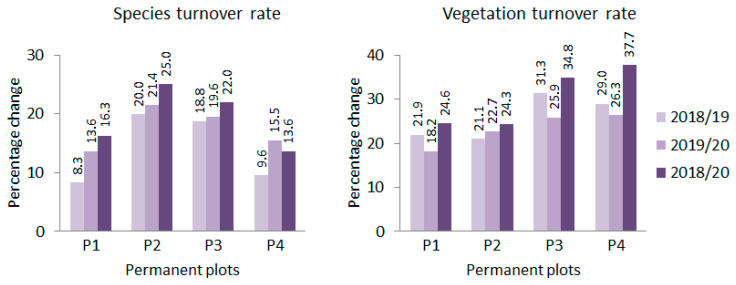
Species and vegetation turnover rates in four permanent plots (P1–P4) of the Natura 2000 loess grassland at “Pócalja”, Central Hungary.

Turnover rates in consecutive years and in two years term for species and vegetation of the plots are shown in Figure 6. Regarding species turnover rates, the values calculated for adjacent years did not differ statistically. Average species turnover rates of plots (*n* = 4) were 14.18% and 17.52% for 2018/2019 and 2019/2020, respectively. However, two-year turnover rates of plots (19.23% in average) proved to be significantly higher (*p* < 0.05) than the one-year rates of 2018/2019, whereas they were identical with that of 2019/2020, according to Friedman’s nonparametric repeated measures ANOVA (Table 2).

Considering vegetation turnover rates, the values calculated for the consecutive years again did not differ statistically. Average vegetation turnover rates of plots (*n* = 4) were 25.83% and 23.28% for 2018/2019 and 2019/2020, respectively. However, two-year turnover rates (2018/2020) of plots (30.35% in average) were significantly higher (*p* < 0.05) than that of 2019/2020, whereas they did not differ from that of 2018/2019, according to the same statistical test used for species turnover rate data (Table 2).

Finally, the species turnover rates of plots calculated between the adjacent years were compared with the corresponding vegetation turnover rates. In the paired *t*-test, vegetation turnover rates proved to be significantly higher (*p* = 0.0067, t = 3.799, d.f. = 7).

## 3. Discussion

Some of the analyses which were performed in this study referred to the stability of the loess grassland in Pócalja, while others referred to its slow successional change. The results indicating stability were obtained mainly from the analyses based on the species data, while changes were observed when vegetation data were taken into consideration.

The share of species among Raunkiaer’s life-form classes and distribution range classes remained practically unchanged during the three years of observation. These indicate that no remarkable textural change of the grassland took place during the study period. The same could be read from the distribution of the species pools of the four plots among the thousand-seed weight classes, which also did not show a significant shift during the three years. As far as the distribution of thousand-seed weight is concerned, it is also necessary to point out that the distribution of the studied loess grassland differed significantly from the distribution observed in the species pool of the entire Hungarian flora [32]. It is known from Salisbury’s studies that the average thousand-seed weight of species coexisting in a habitat is expected to increase as the habitat closes [33,34]. Later, the phenomenon was also demonstrated for the Hungarian flora [35]. In line with this, the maximum values observed for the medium seed weight categories, which characterized the studied grassland, can be assessed as the vegetation in Pócalja being a perfectly developed, considerably closed typical loess steppe grassland. The dominance of species with intermediate thousand-seed weight was also reported for other closed grasslands of north facing slopes [36].

A further sign of maturity of the studied loess grassland is its relatively high Shannon diversity that was, on average, 2.949, 2.809 and 2.812 for 2018, 2019 and 2020, respectively. In comparison, long time uncultivated linear-shaped habitats in verges covered by dry grassland fragments in the Great Hungarian Plain maintained an average diversity of 2.2 [37]. In Transylvania (Romania), for grazed grasslands and hand mowed grasslands, Shannon diversity values were around 2.7–3.2 [38], whereas, for mountainous semi-natural grasslands, diversity values of 2.36–2.68 were reported, depending on various conservation treatments [39]. Beyond local geographical regions, like in Sweden, grazed semi-dry grasslands’ diversity ranged from 2.4 to 3.3 [40], whereas grasslands developed on limestone bedrock maintained diversity of 2.2–3.1 [41]. A slightly higher diversity was reported from the Himalayas, near the border between Pakistan and India, where grazed mountain meadows were characterised by Shannon diversity values of 2.9–3.3 [42].

Besides the signs of stability, some of the analyses indicated gradual change of the studied grassland. The Raunkiaer’s life-form distribution based on species cover values has changed significantly during the three years. The change could be attributed to the gradual decrease in a cover of therophyte (Th+TH) and chamaephyte (Ch) groups, with the simultaneous increase of phanerophytes’ (M) cover. These changes can collectively be explained by the growing prevalence of woody species, as their cover increases competition with therophytes, increasing shading conditions, affecting at the same time both chamaephytes and therophytes. Shrub encroachment is a worldwide phenomenon on mesic or moderately dry grasslands [43,44], and its restrictive effect on annual and biennial species as well as the most light demanding group of perennials has also been documented [45].

Another plant trait which indicates changes among years is the geographical distribution type when species cover data were used. There were fluctuating changes in most of the categories, but a continuous decline was observed in the cosmopolitan (COS) group over the three years. The fluctuations can be explained as the adaptive response of the vegetation to the different climatic years, with which the plant community keeps the grassland closed by increasing or decreasing the cover of individual species according to changes in the prevailing precipitation, irradiance or other regimes [46,47,48]. Similar buffering effect to compensate environmental changes was reported for other grasslands and the phenomenon generally discussed as “the insurance hypothesis” [49,50]. However, the continuous and considerable decrease in the cover of cosmopolitan species can probably be linked with the increased cover of woody species. Most of the species in the cosmopolitan group have more or less ruderal character [51], and are therefore sensitive to increased competition caused by shrub encroachment [52].

In summary, plant trait frequency analyses based on species pool referred more to the stability of the grassland, whereas analyses taking into account the cover of the species indicated a change in the grassland. This result can be interpreted as meaning that the relatively short period of observation did not allow for detecting significant change in species composition, but unidirectional successional change has already occurred in species cover.

Comparing the sensitivity of the examined turnover rates to indicate changes, it is again shown that vegetation turnover rate indicates a larger percentage change than species turnover rate, and the difference between the two types of turnover rates was statistically significant. The study of the exchange rates also provided an additional opportunity to explore the vegetation dynamics processes in the studied loess grassland. According to this, if there is indeed a directional change, the values of the two-year turnover rates should be significantly higher than the values calculated for any of the two cases of one-year turnover rates. However, this was only partially achieved, as for both types of turnover rates the values calculated for the two-year period behaved ambivalently to the one-year rates: in one case, the two-year rate was significantly higher, but not in the other. In this context, it is worth mentioning that considerable differences were also reported among species turnover rates calculated for different pairs of consecutive years in a South Bohemian heathland [53]. Ambiguous results may also be due to climatic fluctuations between years [54]. Mixed signs of fluctuation and slight directional change have also been observed in other grassland studies [55].

In conclusion, the plant trait approach used here proved to be applicable in studying dynamic changes of a species rich loess grassland. Plant traits based on species cover values were more sensitive to changes than traits based on species pool data, at least in the relatively short observation period applied in the present study. Species turnover rates and vegetation turnover rates are also suitable for quantifying dynamic processes in grasslands, but, for detecting directional successional changes, a study longer than three years is advised.

## 4. Materials and Methods

**Figure 7:**
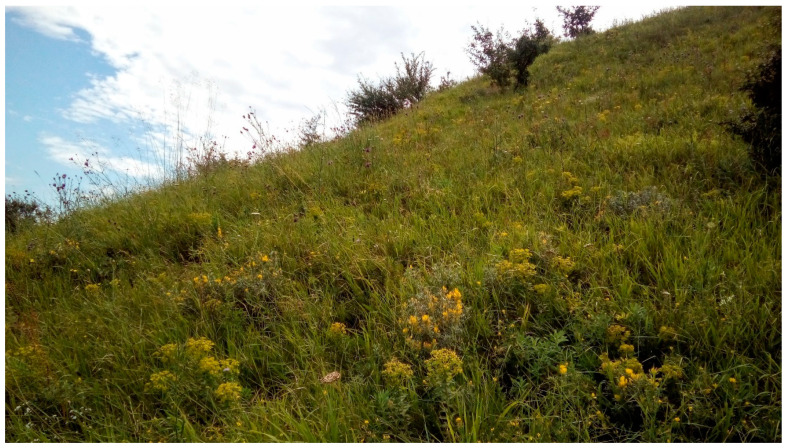
View of the high diversity steppe grassland on loess with *Euphorbia pannonica* and *Chamaecytisus austriacus* in the foreground on 8 July 2019. (Photo: T. Kalapos).

**Figure 8:**
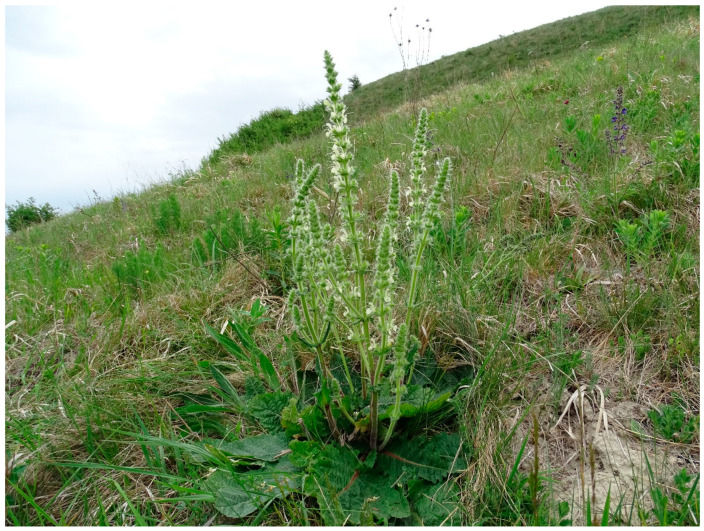
View of the studied steppe grassland on loess with *Salvia austriaca* in the foreground and *S. pratensis* in the upper right corner on 11 May 2020. (Photo: P. Csontos).

*Study site.* Surrounded by intensive agricultural fields (maize, wheat, sunflower), a 14-hectare loess grassland has escaped conversion to arable land at “Pócalja” near the town Bicske (Central Hungary) owing to its relief with steep slopes between 30–50 degrees, facing north, which prevented the use of agricultural machinery (Figure 7 and Figure 8). The grassland is 2 km away from the residential area and is avoided by traffic routes, so human disturbance and trampling practically do not affect the study site. The slope is covered with Haplic Chernozem (loamic) soil according to IUSS Working Group WRB [56], which is characterised by the following physico-chemical properties: pH_H2O_ = 7.1 ± 0.1, pH_KCl_ = 6.8 ± 0.1, C_org_ = 4.00% ± 0.18, N_tot_ = 0.48% ± 0.02, CaCO_3_ content by Scheibler calcimeter = 5.07% ± 2.69, plant available P_2_O_5_ = 104 ± 46.7 and K_2_O = 260 ± 11.0 mg/kg (ammonium-lactate soluble), NH_4_^+^-N = 8.57 ± 0.42 mg/kg, nitrate-N = 1.82 ± 0.56 [57].

The local climatic conditions are characterized by yearly average temperature 9.7–10.0 °C, average temperature in the summer semester 16.0–16.5 °C, yearly average precipitation 550 mm, average precipitation in the summer semester 330 mm. Frosty days can occur from November to April, the average wind speed is around 3 m/s [58].

The protected Natura 2000 area is covered by Pannonic loess steppic grasslands where *Festuca rupicola* and *Brachypodium pinnatum* are the dominant species. Among dicotyledonous species, *Filipendula vulgaris*, *Salvia pratensis*, *Galium verum*, *Chamaecytisus austriacus*, *Euphorbia pannonica* and *Teucrium chamaedrys* attain greater cover values, whereas notable protected, rare herbs are *Echium russicum*, *Adonis vernalis*, *Dianthus pontederae*, *Linum flavum* and *Taraxacum serotinum*.

*Sampling*. Four 4 m by 4 m permanent plots were established in 2018. GPS coordinates of the four plots (P1–P4) are: P1, 47°28.187′ N, 18°39.374′ E; P2, 47°28.193′ N, 18°39.381′ E; P3, 47°28.201′ N, 18°39.386′ E; P4, 47°28.208′ N, 18°39.388′ E, each located at an altitude of about 160 m above sea level. For each plot, vascular plant species and their cover values were recorded by using a percentage scale. Since species asynchrony is a characteristic feature of species rich grasslands [59], surveys of plots were conducted in three occasions (May, July and September) during the growing seasons of years 2018–2020. On every occasion, the species cover was estimated visually by three trained botanists, then their individually estimated values were averaged, thus forming the raw data of the surveys. Plant specimens were identified according to the Hungarian flora [60], plant nomenclature follows [61].

For further analyses, raw data of the three surveys of a certain plot in a year were summarized so that each species was considered with its highest recorded cover value in the growing season, thus forming the phytosociological relevé of the plot. Phytosociological relevés are presented in the Appendix A, Table A1.

*Data analyses*. For each phytosociological relevé, share of species according to Raunkiaer’s life-form categories and distribution range types (chorological types) were calculated [61]. The representativeness of the eight seed weight categories in the species pool of the four plots was also analyzed, based on the thousand-seed weight (TSM) of species [32,36]. (Hereafter, seed denotes all kinds of seed-like disseminules: seed, achene, caryopsis, etc). The following TSM classes were used: 1: ≤0.2 g, 2: 0.21–0.50 g, 3: 0.51–1 g, 4: 1.01–2 g, 5: 2.01–4 g, 6: 4.01–10 g, 7: 10.1–50 g, 8: >50 g. Where appropriate, the plant trait spectra were subjected to homogeneity tests among the three studied years.

Species-abundance diversity of the plots was calculated based on the Shannon–Wiener formula, with natural base of logarithm.

For each permanent plot, species turnover rate (T_sp_) was calculated between years 2018 and 2019, as well as years 2019 and 2020, according to the formula:

T_sp_ = (I + D)/(I + D + 2U) ∗ 100

where I is the “increases”: the number of species occurred in the second year of the comparison but were absent in the first year; D is the “decreases”: the number of species occurred in the first year of the comparison but disappeared to the second year; U is “unaltered”: the number of common species of the two years. In this way, the results are given in a percentage form [19,62].

Vegetation turnover rate (T_veg_) between two subsequent years were also calculated for each permanent plot according to the formula:

$$T_{\mathrm{Veg}(y1)(y2)}=\frac{\sum_{i=1}^{n}\left|x_{i\left(y1\right)}-x_{i\left(y2\right)}\right|}{\sum_{i=1}^{n}\left(x_{i\left(y1\right)}+x_{i\left(y2\right)}\right)}*100$$

where *x*_*i*(*y*1)_ and *x*_*i*(*y*2)_ is the percentage cover of species *i* in the first year and in the subsequent year, respectively, and *n* is the cumulative number of species registered in the given permanent plot during year 1 and year 2. Thus, for a given species, whether its cover increases or decreases compared to the previous year, it contributes to increasing the value of the vegetation turnover rate.

Additionally, a two-year turnover rate (2018/2020) was also calculated to detect changes in the flora and in the vegetation.

A paired sample *t*-test was used to compare species- and vegetation turnover rates between 2018/2019 and 2019/2020. Friedman’s nonparametric repeated measures ANOVA followed by Dunn’s multiple comparison post hoc test was used for comparisons for more than two data sets.

PAST-3.25 and InStat-3.06 packages were used to perform statistical tests [63,64].

## Figures and Tables

**Table 1:** The Shannon–Wiener diversity of the four permanent plots (P1–P4) in the “Pócalja” Nature-2000 loess grassland (Central Hungary), during three consecutive years.

	2018	2019	2020
P1	3.0426	2.8841	2.7083
P2	2.7015	2.4605	2.6303
P3	2.8729	2.9251	2.8833
P4	3.1803	2.9649	3.0256

**Table 2:** Results of statistical tests on species- and vegetation turnover rates according to Friedman’s nonparametric repeated measures ANOVA followed by Dunn’s multiple comparisons test. (* = indicates significant differences, ns = not significant).

Compared Turnover Rates	Rank Sum Difference	*p*-Value	Significance
species turnover			
2018/19 vs. 2019/20	−5.0	*p* > 0.05	ns
2018/19 vs. 2018/20	−7.0	*p* < 0.05	*
2019/20 vs. 2018/20	−2.0	*p* > 0.05	ns
vegetation turnover			
2018/19 vs. 2019/20	+2.0	*p* > 0.05	ns
2018/19 vs. 2018/20	−5.0	*p* > 0.05	ns
2019/20 vs. 2018/20	−7.0	*p* < 0.05	*

## Data Availability

Data are contained within the article and its Appendix A.

## Abbreviations

RLF	Raunkiaer’s life forms
Th	therophyte
TH	hemitherophyte
H	hemicryptophyte
G	geophyte
Ch	chamaephyte
N	nanophanerophyte
M	microphanerophyte
FLE	floristic element (chorological type)
SW	seed weight category
na	not available

## Appendix A

**Table A1:** List of species with their cover values in the Natura-2000 loess grassland at “Pócalja”, Central Hungary, in the years 2018–2020.

Plots	Plot-1			Plot-2			Plot-3			Plot-4			RLF	FLE	SW
Years	2018	2019	2020	2018	2019	2020	2018	2019	2020	2018	2019	2020			
Species Name															
*Achillea collina* J. Becker			2.0			0.1			1.0			1.0	H	CON	1
*A. pannonica* Scheele	2.0	1.0	0.1	0.5	1.0	0.1	2.0	5.0	1.0	3.0	1.0	0.5	H	PON	1
*Adonis vernalis* L.	2.0	1.0	2.0	4.0	1.0	3.0	4.0	3.0	4.0	3.0	2.0	2.0	H	CON	7
*Agrimonia eupatoria* L.				0.1			1.0	1.0	2.0	2.0	3.0	3.0	H	EUR	7
*Agropyron intermedium* Host					0.1	9.0		0.1	10.0	2.0	15.0	14.0	G	PoM	6
*A. repens* (L.) P. B.				0.1			1.0	5.0	0.1	3.0	10.0		G	CIR	5
*Ailanthus altissima* (Mill.) Swingle						0.1							MM	ADV	7
*Ajuga reptans* L.										0.1			H-Ch	EUR	4
*Anthericum ramosum* L.	1.0												G	CEU	6
*Arabis hirsuta* (L.) Scop.	0.1	0.1	0.1							0.1	0.5	0.1	TH-H	CIR	1
*Arrhenatherum elatius* (L.) J. et C. Presl	0.1			1.0			0.1		0.5				H	EUA	5
*Asperula cynanchica* L.			0.1							0.5	1.0	1.0	H	PoM	3
*Astragalus austriacus* Jacq.										1.0	0.5	0.5	H	CON	3
*A. onobrychis* L.	1.0	1.0	0.1				1.0	0.5	0.5				H	CON	4
*Betonica officinalis* L.	0.1	0.5	0.1										H	EUA	4
*Bothriochloa ischaemum* (L.) Keng			0.5						0.5	0.1	0.1	1.0	H	PoM	2
*Brachypodium pinnatum* (L.) P. B.	19.0	30.0	30.0	40.0	45.0	30.0	9.0	20.0	25.0	25.0	25.0	13.0	H(Ch)	EUA	6
*Brassica elongata* Ehrh.										0.1			TH-H	CON	2
*Briza media* L.	2.0	2.0	0.5	0.1						3.0	0.1	1.0	H	KOZ	3
*Bromus erectus* Huds.				0.1			0.1						H	PaB	6
*B. inermis* Leyss.									0.1	5.0	1.0	8.0	H	CIR	5
*Campanula bononiensis* L.	0.1					0.1	2.0	0.5	0.1				H	EUA	1
*C. glomerata* L.				2.0	0.1							0.1	H	EUA	1
*Carduus acanthoides* L.		0.1											TH	EUR	4
*Carex humilis* Leyss.	5.0	3.0	2.0	0.1	0.1		0.1			0.5	1.0	0.5	H	CON	4
*C. michelii* Host	1.0	1.0	0.5	2.0	5.0	3.0	38.0	15.0	13.0	0.1	0.1	1.0	H	SMO	5
*C. tomentosa* L.	0.1	0.1	0.1	0.1	0.1		0.1			0.1	0.1	0.1	G	EUA	4
*Carlina vulgaris* L.	0.1	0.5	0.1									0.1	TH-H	EUA	4
*Centaurea micranthos* S.G. Gmel.				0.1					0.1				TH-H	PON	4
*C. pannonica* (Heuff.) Simk.	1.0	0.5	1.0	2.0	4.0	4.0	0.5	1.0	2.0	3.0	2.0	5.0	H	PoP	4
*C. sadlerana* Janka	3.0	5.0	7.0										H	PAN	6
*Chamaecytisus austriacus* (L.) Link	10.0	10.0	13.0	8.0	6.0	5.0	2.0	5.0	3.0	0.1	0.1	0.5	N	PoP	6
*Chrysanthemum leucanthemum* L.										0.1		0.1	H	EUA	2
*Cirsium arvense* (L.) Scop.	1.0	2.0	0.1										G	EUA	4
*Clinopodium vulgare* L.										9.0	3.0	1.0	H	CIR	2
*Cornus sanguinea* L.	0.1	0.1	0.1	0.1					0.1	0.1	0.1	0.1	M	SME	8
*Coronilla varia* L.							0.1	0.1					H	PoM	5
*Crataegus monogyna* Jacq.	0.1	1.0	0.5	2.0	2.0	3.0	2.0	2.0	3.0	1.0	1.0	3.0	M	EUR	7
*Dactylis glomerata* L.	1.0	2.0	0.5	2.0	1.0		0.1		0.1	8.0	2.0	0.5	H	KOZ	3
*Daucus carota* L.			0.1				0.1	0.5		1.0		0.1	Th-TH	KOZ	5
*Dianthus pontederae* Kern.	0.1	0.5	0.1	0.1	0.5	0.1	0.1	0.1	0.1	0.1		0.1	H	PAN	3
*Dorycnium germanicum* (Gremli) Rikli							3.0	1.0	2.0				Ch	ALB	5
*Eryngium campestre* L.	2.0	0.5	1.0	4.0	3.0	3.0	1.0	2.0	2.0	1.0	1.0	1.0	H	PoM	5
*Euphorbia pannonica* Host	10.0	14.0	15.0	8.0	4.0	7.0	15.0	20.0	14.0	6.0	5.0	5.0	H	PaB	4
*Falcaria vulgaris* Bernh.							1.0	0.5	0.1	1.0	0.1	0.1	Th-TH	EUA	4
*Festuca rupicola* Heuff.	25.0	35.0	25.0	20.0	25.0	25.0	10.0	20.0	25.0	20.0	30.0	35.0	H	EUA	3
*Filipendula vulgaris* Mönch	15.0	10.0	8.0	8.0	11.0	5.0	15.0	12.0	12.0	15.0	12.0	10.0	H	EUA	3
*Fragaria viridis* Duch.	0.1	0.5	0.1	1.0	1.0	0.5	2.0	3.0	2.0	3.0	3.0	3.0	H	CON	2
*Galium aparine* L.	0.1												Th	KOZ	6
*G. glaucum* L.	1.0	1.0	0.1	1.0	1.0	1.0	0.1	0.1	0.1	0.1	0.5	1.0	H	PoM	4
*G. verum* L.	7.0	8.0	5.0	6.0	15.0	5.0	5.0	15.0	6.0	5.0	10.0	2.0	H	EUA	3
*Helictotrichon praeustum* (Rchb.) Tzvelev	1.0	1.0	0.5		0.5	2.0	6.0	5.0	10.0	3.0		2.0	H	EUR	na
*Helictotrichon pubescens* (Huds.) Pilger	1.0	1.0	0.1	1.0	2.0	1.0	1.0	0.1	0.5	5.0	0.1	1.0	H	EUA	5
*Hieracium cymosum* L.	0.1	0.5	0.1									0.1	H	EUA	na
*H. sabaudum* L.										0.1		0.1	H	CEU	2
*Inula sp*.			0.1				0.1						H	CON	na
*Knautia arvensis* (L.) Coult.	1.0	2.0	2.0	1.0	1.0	1.0	2.0	1.0	0.5	1.0	1.0	1.0	H	EUA	5
*Koeleria cristata* (L.) Pers.	1.0	1.0	0.1			0.1	0.1						H	KOZ	2
*Lathyrus tuberosus* L.				0.1		0.1							H-G	EUA	7
*Leontodon hispidus* L.	0.1	0.1	0.1			0.1							H	EUR	4
*Ligustrum vulgare* L.						0.1							M	AsM	7
*Linum catharticum* L.	0.1	0.1	0.1										Th(H)	EUR	1
*L. flavum* L.	0.1	0.5	1.0				0.1					0.5	H	PoP	3
*Lotus corniculatus* L.	1.0	0.1	0.1	1.0	0.5	0.5				1.0	0.1	0.1	H	EUA	4
*Luzula campestris* (L.) Lam. et DC.	0.1	0.1											H	KOZ	3
*Medicago falcata* L.	3.0	0.1	0.5	5.0	2.0	2.0	5.0	2.0	3.0	3.0	5.0	2.0	H	EUA	4
*M. lupulina* L.	0.1												Th-TH	EUA	4
*Melilotus officinalis* (L.) Pall.			0.1	0.1		0.1			0.1			0.1	Th-TH	EUA	4
*Muscari comosum* (L.) Mill.				0.1		0.1							G	SME	6
*M. racemosum* (L.) Mill.	0.1												G	SME	5
*Ononis spinosa* L.				1.0	1.5	1.0	0.5	0.5	0.5				H-Ch	EUR	5
*Ornithogalum kochii* Parl.	0.1	0.5			0.1						0.1		G	PoM	na
*Orobanche teucrii* Holandre	0.1	0.1											G	CEU	1
*Phleum phleoides* (L.) Karsten	0.1	0.1	0.1				0.1		0.1				H	CON	1
*Picris hieracioides* L.	0.1	0.5	2.0	0.1	0.1	0.1	0.1	1.0		0.1	2.0	1.0	TH-H	EUA	3
*Pimpinella saxifraga* L.	0.1		0.1	0.1	0.5	0.1	0.5	0.5	0.5	0.5	0.1	2.0	H	EUA	3
*Plantago media* L.	1.0	0.5	0.5							0.5	0.1	0.1	H	EUA	2
*Poa pratensis* L.	1.0	1.0	0.1	8.0	0.5	0.5	2.0		1.0	0.1	1.0		H	KOZ	2
*Prunella grandiflora* (L.) Scholler	0.1	0.1	0.5					0.1	0.1				H	EUR	3
*P. laciniata* (L.) Nath.	0.1	0.5	0.1						0.1				H	SME	4
*P. x bicolor* Beck	0.1		0.1				1.0	0.5	1.0	0.1			H	SME	na
*Prunus cerasifera* Ehrh.						0.1		0.1				0.1	M	ADV	8
*P. spinosa* L.	0.1	0.1	0.5	0.1	0.1	0.5	0.1	0.5	0.5	4.0	8.0	13.0	M	EUR	8
*Ranunculus polyanthemos* L.	1.0	0.5	0.1	0.1	0.1	0.1				2.0	1.0	0.5	H	PON	5
*Rhamnus catharticus* L.	0.1	0.1	0.1	0.1	0.1	0.1				0.1	0.1	0.1	M	EUA	7
*Salvia nemorosa* L.				0.1	0.5	0.5							H	EUR	3
*S. pratensis* L.	3.0	8.0	7.0	4.0	5.0	3.0	5.0	10.0	6.0	4.0	7.0	6.0	H	EUR	4
*Sanguisorba minor* Scop.			0.1										H	EUR	6
*Scabiosa ochroleuca* L.	1.0	0.1	0.1					0.1		1.0	0.1	1.0	H	CON	4
*Senecio jacobaea* L.			0.1							0.1	0.1	0.1	H	EUA	2
*Seseli annuum* L.	1.0	0.1	0.1	0.1	0.1	0.1	0.5	0.1	0.1	0.1	0.1	0.5	Th-TH-H	CON	2
*Setaria viridis* (L.) P. B.							3.0			0.1			Th	EUA	3
*Stachys recta* L.	0.1			0.1			0.1						H	PoM	4
*Taraxacum officinale* Weber			0.1	0.1	0.1							0.1	H	EUA	2
*T. serotinum* (W. et K.) Poir.	0.5	0.5	0.5							1.0	2.0	2.0	H	PoP	3
*Teucrium chamaedrys* L.	8.0	8.0	8.0	3.0	3.0	2.0	15.0	15.0	6.0	10.0	8.0	5.0	Ch	SME	4
*Thesium linophyllon* L.	0.1	0.1	0.1	0.1		0.1				0.1	0.1	0.1	G-H	CEU	5
*Thlaspi perfoliatum* L.	0.1	0.1											Th	SME	2
*Thymus pannonicus* All.	1.0	5.0	0.1	0.1			1.0	1.0		2.0	3.0	0.5	Ch	CEU	na
*Tragopogon orientalis* L.	1.0	2.0	0.1				0.1	0.5	0.1				TH-H	EUA	6
*Trifolium alpestre* L.	0.1						0.1	3.0	1.0	0.1	0.1		H	CEU	5
*Trifolium montanum* L.	1.0	3.0	1.0	3.0	1.0	1.0	2.0	5.0	1.0	1.0	2.0	1.0	H	CON	3
*Verbascum phoeniceum* L.	0.5	1.0	0.5							0.1	1.0	0.5	H	PON	1
*Veronica austriaca* L.	0.1	0.1	0.1					0.1					H	PON	1
*Veronica spicata* L.	0.5	0.1	0.1	0.1		0.1	0.1			0.5	0.5	0.1	H-Ch	EUA	1
*Viola canina* L.				0.1						0.1			H	EUA	3
indet. dicot. 1			0.1												
indet. dicot. 2												0.1			
**Number of species**	**71**	**62**	**69**	**51**	**39**	**45**	**52**	**44**	**48**	**62**	**53**	**63**			
**Total cover (%)**	**140.8**	**169.0**	**141.9**	**141.0**	**144.6**	**120.4**	**160.9**	**178.5**	**161.5**	**162.8**	**172.8**	**155.1**			
